# Revealing the Mechanisms of Alzheimer’s, Parkinson’s and Huntington’s Diseases Through Invertebrate Models

**DOI:** 10.3390/biology15161351

**Published:** 2026-08-10

**Authors:** Xing Ding, Peijin Wang, Limin Zhou, Xingxia Li

**Affiliations:** 1 College of Life Sciences, Neijiang Normal University, Neijiang 641100, China; 18892751967@163.com (L.Z.); 13698308872@163.com (X.L.); 2 Key Laboratory of Regional Characteristic Agricultural Resources, Department of Education, Si-Chuan 641100, China; 3 College of Horticulture and Landscape Architecture, Southwest University, Chongqing 400715, China; wangpj25@email.swu.edu.cn

**Keywords:** Alzheimer’s disease, Parkinson’s disease, Huntington’s disease, conservative mechanism, invertebrate models, inspiration for the future

## Abstract

The neural circuits of the human brain are highly complex (due to the number of neurons and the diversity of synaptic connections), hindering the analysis of the pathological mechanisms of neurodegenerative diseases. Invertebrates with simple yet well-differentiated nervous systems have a natural advantage over mammalian model organisms in the identification of pathogenic genes and functional studies of neurodegenerative diseases. They can provide unique and profound insights into the pathogenesis of complex human neurodegenerative diseases and the formulation of intervention strategies. This article reviews the conserved mechanisms of three neurodegenerative diseases across species, including protein homeostasis imbalance and aggregation toxicity, mitochondrial dysfunction and metabolic abnormalities, axonal transport defects, and loss of synaptic function. Based on research on three invertebrates in the field of neurodegeneration, namely *Caenorhabditis elegans* (*C. elegans*), *Drosophila melanogaster* (*D. melanogaster*), and *Bombyx mori* (*B. mori*), we cover three major types of neurodegenerative diseases: Alzheimer’s disease (AD), Parkinson’s disease (PD), and Huntington’s disease (HD). The aim is to find important inspirations for the future prevention and treatment of neurodegenerative diseases from the aspects of the material basis and existing treatment strategies.

## Simple Summary

This article reviews the conserved mechanisms underlying the occurrence of three neurodegenerative diseases across species, with a focus on major studies involving three invertebrate models. It highlights the research history, key milestones, critical aspects, and significant achievements related to neurodegenerative diseases in these models, aiming to identify potential insights for future research on neurodegenerative diseases from various perspectives.

## 1. Introduction: The Significant Position of Invertebrate Models in Research on Neurodegenerative Diseases

### 1.1. From Complex Brains to Simplified Systems

Data from the United Nations Population Division show that the global trend of aging populations is accelerating, with a significant increase in the number and proportion of the elderly population. In 1980, the global elderly population was 258 million. By 2022, the number of people aged 65 and above had reached 771 million, and it is projected to increase to 1.6 billion by 2050 [1]. Simultaneously, the incidence of aging-related diseases is rising. Currently, aging and its associated diseases have become a global public health issue that transcends national boundaries; neurodegenerative diseases are particularly prominent. In 2021, more than one-third of the global population was affected by a neurological disease [2]. Such a large group of patients stems from the high precision and fragility of the extremely complex neural circuits in the human brain. The neural circuits in the human brain are highly complex. They are characterized by a scale of orders of magnitude; that is, the brain contains approximately 8.6 × 10^10^ neurons that form a precise interconnecting network including over 10^14^ synapses. There are also heterogeneous characteristics in the functional dimension. The dynamic plasticity of synaptic connection strength, the spatiotemporal specificity of neurotransmitter release, and the multi-dimensional interactivity of signaling pathways jointly endow this network with extremely strong nonlinear regulatory capabilities [3,4]. This obstructs the analysis of the pathological mechanisms of neurodegenerative diseases. Although invertebrates are relatively simple in the evolutionary hierarchy, they have natural advantages. These organisms possess a large number of genes that are directly homologous to those of humans [5,6,7]. The discovery and functional clarification of these direct homologous genes have opened up new avenues for scientists to study the mechanisms of human diseases in invertebrate models. Moreover, they have relatively simple biological structures but possess complete and fully functional physiological and biochemical reaction systems, including regulation of the nervous system, the operation of metabolic pathways, and the synergy of various biochemical reactions [8,9,10]. Although the nervous systems of different species exhibit marked differences in structural complexity and functional capacity, many fundamental cellular biological processes—including protein homeostasis regulation, mitochondrial energy metabolism, and synaptic signal transduction—are evolutionarily conserved across species from invertebrates to humans [11,12,13,14]. While these conserved pathways are broadly shared, their regulatory mechanisms and functional outputs may vary across species. However, converging evidence indicates that the dysregulation of these conserved processes is closely linked to the occurrence and development of neurodegenerative diseases [15,16,17]. Therefore, invertebrate models are of extreme significance in revealing the mechanisms of neurodegenerative diseases.

To ensure comprehensive coverage, we systematically searched PubMed, Web of Science, Scopus, Embase, and Google Scholar using keyword combinations for each species and disease, including terms such as “*Caenorhabditis elegans* (*C. elegans*),” “*Drosophila melanogaster* (*D. melanogaster*),” “*Bombyx mori* (*B. mori*),” “Alzheimer’s disease (AD),” “Parkinson’s disease (PD),” “Huntington’s disease (HD),” “neurodegenerative diseases,” and their synonyms (e.g., “α-synuclein,” “polyQ,” and “β-amyloid protein (Aβ)”). We included original experimental studies, reviews, and authoritative articles focusing on molecular mechanisms, genetic regulation, and therapeutic interventions in these three invertebrate models.

### 1.2. Comparison of the Advantages of the Three Models

At present, three experimental animals have made significant contributions in this field, namely *C. elegans*, *D. melanogaster*, and *B. mori*. These animals have a long history of research and use as invertebrate models in modern biology. *C. elegans* was introduced by Brenner in the 1960s as a model for developmental and neurobiological studies. *D. melanogaster* was first brought into genetics research by Morgan in the early 20th century and has since accumulated over a century of experimental history. *B. mori* is an economically important insect with over 5000 years of domestication that has been progressively adopted in scientific research since the 19th century. Following the recent sequencing of its genome, it has emerged as a novel invertebrate model. Over a century of systematic research in modern physiology and genetics has established a mature set of breeding norms and genetic manipulation systems for each of these three species. On this basis, inter-laboratory genetic background variations for the same strain are relatively controllable. Research shows that the lack of genetic background control is a major factor affecting the reproducibility of experimental results [18]. *C. elegans*, *D. melanogaster*, and *B. mori*—owing to their clearly defined strain provenance and standardized rearing conditions—may offer advantages in genetic background stability, thereby enhancing the comparability and reproducibility of experimental data.

Of these models, *C. elegans* has two unique biological characteristics. The first is a constant cell lineage (there are 959 somatic cells in hermaphroditic adults, and the developmental trajectory is completely traceable) [19,20,21]. The second is the fully resolved connectome of all animals (including 302 neurons, 6393 chemical synapses, and 890 gap junctions) [22,23], coupled with its overall transparency and extremely short life cycle (about 2–3 weeks) [24]. In addition, there is substantial overlap in biological processes between *C. elegans* and mammals, underscoring the translational conservation of fundamental pathological mechanisms [25]. Furthermore, the genetic regulation of programmed cell death and RNA interference was initially elucidated in *C. elegans* [24,26,27,28]. Therefore, *C. elegans* has become an ideal model for the study of cell fate determination, programmed apoptosis, and conserved signaling pathways. *D. melanogaster* is characterized by its strong reproductive capacity (a healthy female can lay hundreds of eggs), relatively short life cycle (about 10 days), and complex organ systems (such as specialized structures like compound eyes and wing veins). The regulatory mechanisms of its embryonic development and organ formation are highly conserved among mammals (such as the homology between the midgut and the human small intestine), and it has transcription factors similar to those in humans [29,30]. Moreover, several important pathways related to normal development were initially identified in *D. melanogaster* [28,31]. More importantly, the developmental process of *D. melanogaster* has been precisely analyzed. The central nervous system of *D. melanogaster* embryos is composed of approximately 15,000 neurons and glial cells, which develop and are produced in about one day [32]. Neural stem cells (neuroblasts) produce differentiated offspring while self-renewing through asymmetric division [33]. The differentiation of neurons is closely related to the regulation of three key mechanisms: spatial modeling (determining regional identity), temporal modeling (determining birth order identity), and terminal selection of transcription factors (maintaining terminal characteristics) [34]. Neuroblasts sequentially express a series of transcription factors such as Hunchback, Kruppel, Pdm, and Castor, meaning that neurons produced at different times have different fates [33]. Furthermore, by targeting the expression of mutant human pathogenic proteins [such as Aβ, α-synuclein, and mutant huntingtin protein (HTT)], *D. melanogaster* models can reproduce key pathological features including selective neuronal degeneration, protein aggregate formation, and behavioral dysfunction [35]. Their advantage lies in the fact that the *D. melanogaster* brain can allow genetic screening in relatively simple neural structures, identification of disease-modifying genes, and preclinical efficacy evaluation of potential therapeutic compounds [36]. Therefore, *D. melanogaster*, with its powerful genetic system, precise analysis of the neuronal development process, and short generation cycle, has become a classic model system for studying neurodegenerative diseases such as AD, PD, and HD [37]. As a representative species of *Lepidoptera* insects, *B. mori* has a relatively large individual size. Its unique advantages lie in its specialized silk gland organs (the posterior silk glands efficiently synthesize silk fibroin) and larger individual size [38]. *B. mori* precisely regulates the spatiotemporal specificity of silk protein synthesis using transcription factors such as Bmsage and Bmdimm in its silk gland cells [39,40]. In addition, *B. mori* also has other characteristics, such as a clear genetic background and distinct boundaries between different stages of development [41]. Therefore, *B. mori* is an ideal system for understanding the regulation of protein synthesis, immune responses, and pathological mechanisms.

**Figure 1:**
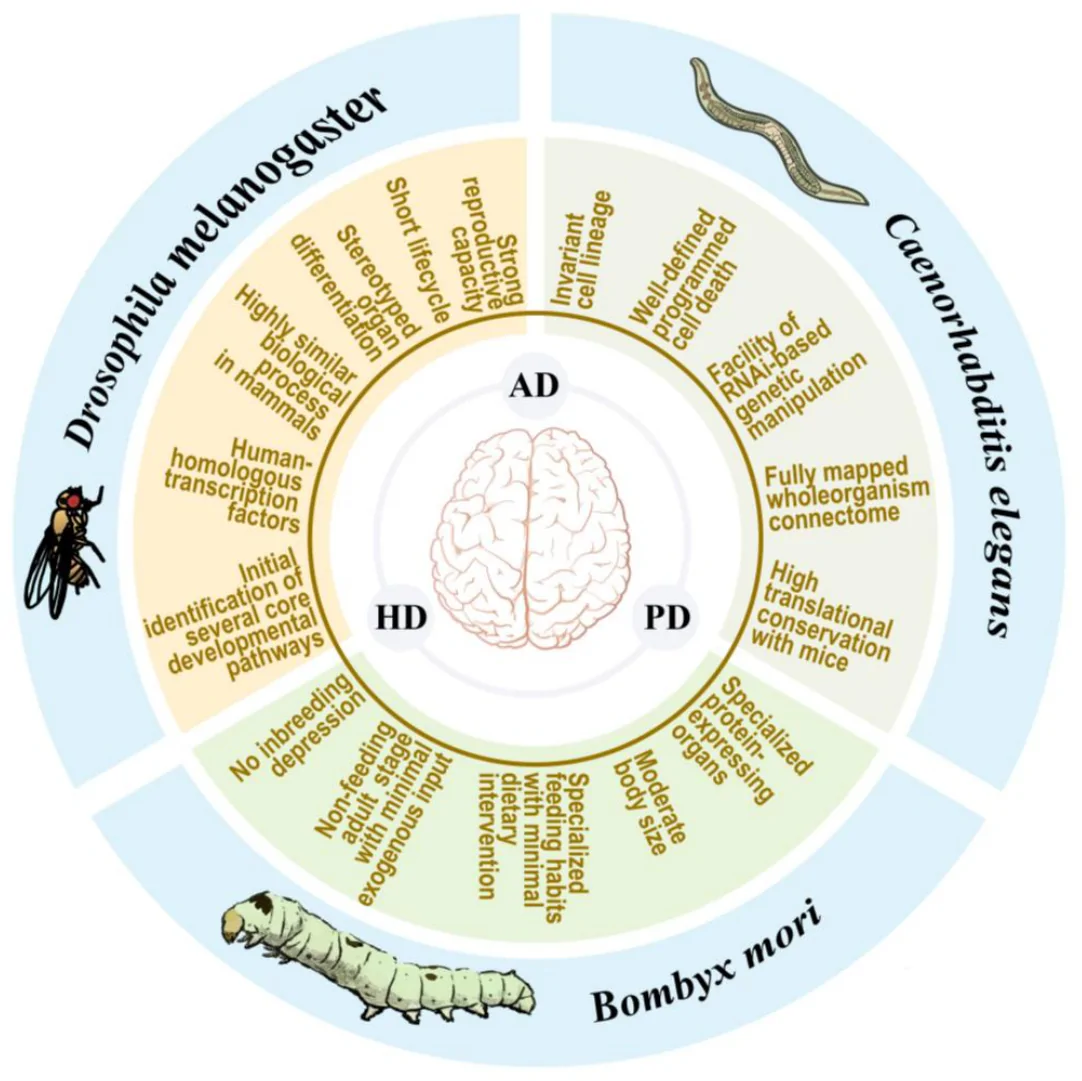
Summary of the advantages of three invertebrate models.

In conclusion, among the three model organisms selected, *C. elegans* has a nervous system with only 302 neurons and a relatively fixed structure, which is suitable for analyzing the fundamental logic of sensory–motor conversion; *D. melanogaster* is equipped with a wealth of genetic tools, and analysis of its developmental process is more precise, making it suitable for studying the modular organization of more complex behavioral regulation; and the neurons of *B. mori* are relatively large in size, and the ganglia are relatively clear, providing a unique anatomical advantage for subcellular morphological tracking. These three invertebrate models each have their own strengths and complement each other’s advantages, which can establish a multi-dimensional research system for neurodegenerative diseases (Figure 1).

Unlike vertebrate studies, which are subject to stringent ethical oversight, invertebrate models such as *C. elegans*, *D. melanogaster*, and *B. mori* face considerably lighter regulatory restrictions. This reduced ethical burden permits larger sample sizes, more aggressive/interventional experimental designs, and truly high-throughput screening—factors that directly enhance statistical power and cross-laboratory reproducibility [42,43]. Meanwhile, some limitations should be noted when using invertebrate models for neurodegenerative disease research. These models have limited behavioral complexity and cannot replicate human cognition. The lack of orthologs for certain disease-related genes and species-specific signaling differences may affect modeling accuracy. Short lifespans, while beneficial for high-throughput screening, do not reflect the decades-long progression of human diseases. Additionally, the inability to model late-stage complications limits their use in studying the full disease course and in translational research [44,45].

### 1.3. The Main Content and Objectives of This Article

This review aims to elucidate the complementary value of three invertebrate models in dissecting the evolutionarily conserved mechanisms of three neurodegenerative diseases. Most reviews focus on a single model. Those on *C. elegans* and *D. melanogaster* models typically summarize their contributions to the identification of molecular targets in neurodegenerative diseases. Studies on *B. mori*, in contrast, have largely concentrated on the neuroprotective activities of its fibroin and sericin or on the establishment of novel PD models. Few reviews have integrated *B. mori* with *C. elegans* and *D. melanogaster* within the same framework to systematically compare their respective advantages and complementarity in studying multiple neurodegenerative diseases (as outlined in Section 1.2.). Incorporating *B. mori* into the discussion of mainstream model organisms thus represents a valuable supplement to the existing review landscape. While *C. elegans* and *D. melanogaster* are primarily used as disease models for drug target discovery and compound screening, *B. mori* can serve not only as a disease model but also as a direct source of therapeutic materials. The core value of this review lies in constructing a comprehensive view that extends from “individual points” to a “broader landscape.” Moreover, compared with previous reviews, this work places additional emphasis on the research trajectories and key milestones in neurodegenerative disease studies across the three models, which distinguishes it from the foci of other reviews.

## 2. Core Foundations: Conserved Neurodegenerative Mechanisms Across Species

### 2.1. Protein Homeostasis Imbalance and Aggregation Toxicity

In the core mechanisms of neurodegenerative diseases, the imbalance of protein homeostasis plays a crucial role. The protein homeostasis network within cells is responsible for maintaining the correct synthesis, folding, transport, and degradation of proteins. The dynamic balance of proteins is crucial for cell survival, tissue function, and the health of the organism. The maintenance of protein homeostasis in eukaryotic cells is mainly accomplished through two pathways and their mutual cooperation. One is the ubiquitin–proteasome system (UPS), and the other is the autophagy–lysosome pathway (ALP) [46]. UPS is regarded as the preferred approach to eliminating unfolded or misfolded proteins (UFP/MFP). Firstly, ubiquitin is attached to the target protein through ubiquitin-activating enzyme E1, ubiquitin-conjugating enzyme E2, and ubiquitin ligase E3 to ubiquitinate the target protein. Then, the target protein is degraded with the proteasome as the core site, and the resulting product can be recycled. ALP forms autophagic lysosomes, which utilize the degradation effect of lysosomes to eliminate abnormal proteins and organelles, thereby preventing the accumulation of toxic aggregates. When lysosomal function is impaired, the activity of acidic hydrolases within it decreases, leading to the formation of aggregates of autophagy substrates (such as misfolded proteins and damaged organelles) mediated by p62/SQSTM1 and their selective capture by autophagy, but they cannot be effectively degraded, thus causing p62 to accumulate. At this moment, SQSTM1/p62 plays a dual role as a key mediator for cargo selection and an autophagic matrix. Through the restoration of its dependent protein inhibition, lysosomal function can be repaired to reactivate the autophagic flow, enabling clearance of the P62–substrate complex [47]. In contrast, the general upregulation of autophagy is a broad enhancement of autophagy initiation (such as through mTOR inhibition) in response to nutritional deficiency or stress, but it is not necessarily accompanied by targeted repair of lysosomal function. UPS and ALP are closely related and work in synergy to jointly maintain protein homeostasis in eukaryotic cells. When this precise system is imbalanced, misfolded or unfolded proteins will abnormally accumulate, forming oligomers or even insoluble fibrous aggregates. These aggregates not only lose their normal functions themselves but also interfere with key cellular processes such as synaptic transmission, mitochondrial function, and axonal transport through “acquired toxicity”, ultimately inducing neuronal dysfunction and death. This cascade reaction from homeostasis imbalance to aggregated toxicity is the common pathological basis of many neurodegenerative diseases.

Studies using classic model organisms such as *C. elegans*, *D. melanogaster*, and *B. mori* have provided a wealth of intuitive evidence for our understanding of this mechanism. As one of the neurodegenerative diseases that has received the most attention, AD is mainly characterized by the deposition of extracellular Aβ and neurofibrillary tangles formed by intracellular hyperphosphorylated Tau. In *C. elegans*, by taking advantage of the transparent nature of their bodies, researchers can directly observe the aggregation process of Aβ in muscles or neurons. Overexpression of this protein in *C. elegans* can cause a significant decline in motor ability, a decrease in reproductive capacity, and defects in learning and memory ability. This directly demonstrates the destructive impact of protein aggregation on cellular function, and the key homeostatic genes regulating this process can be screened out using sophisticated genetic tools in *C. elegans* [48]. In *D. melanogaster*, researchers were able to precisely simulate the pathological features of PD in the brain by expressing human mutant α-synuclein. The aggregation of these exogenous proteins can lead to progressive loss of dopaminergic neurons and cause *D. melanogaster* to exhibit phenotypes similar to PD, such as movement disorders (including decreased climbing ability), clearly demonstrating the toxic effects of specific protein aggregation on neurons [49]. Enhancing mitochondrial protein homeostasis can delay and inhibit the aggregation and toxicity of Aβ in transgenic models of AD (including cells, *C. elegans*, and mice) [50]. In the *D. melanogaster* model of AD, enhanced sleep can improve synaptic integrity and function, regulate protein homeostasis, and slow neurodegenerative progression [51]. The naked pupa (Nd) mutant of *B. mori* was reported to exhibit a series of molecular characteristics, such as abnormal accumulation of p62 and ubiquitinated proteins, which are reminiscent of the protein aggregation-related phenotype observed in human neurodegenerative diseases [52]. This result has opened up a new door for neurodegenerative disease research in *B. mori*.

### 2.2. Mitochondrial Dysfunction and Metabolic Abnormalities

It is well known that mitochondria are the “energy factories” of cells and are involved in many important functions of cells [53]. Neurons, as highly energy-consuming cells, rely heavily on ATP produced by mitochondria to maintain their key functions such as excitability, synaptic transmission, and axonal transport. When mitochondrial function is impaired, it first leads to a crisis in cellular energy (ATP) supply, directly affecting the electrophysiological activities and survival of neurons. Secondly, dysfunctional mitochondria can produce excessive reactive oxygen species (ROS), triggering oxidative stress and damaging lipids, proteins, and DNA. In addition, mitochondria are also responsible for regulating intracellular calcium ion homeostasis. Their dysfunction can lead to calcium overload and further activate the apoptotic pathway. These abnormalities form a vicious cycle: insufficient energy and oxidative stress will exacerbate protein misfolding and aggregation (such as Aβ and Tau proteins), and these pathological protein aggregates will in turn damage mitochondria, ultimately and irreversibly leading to neuronal apoptosis. Therefore, targeting mitochondrial function and brain energy metabolism has become a highly promising neuroprotective strategy.

Studies conducted using model organisms such as *C. elegans*, *D. melanogaster*, and *B. mori* have provided strong evidence for our understanding of this process. Mitochondrial complex I is important for cellular ATP production by transporting electrons and generating proton gradients on the inner mitochondrial membrane [54]. Researchers have clarified the function of complex I in mitochondrial respiration from biochemical, cell biological, and structural aspects, respectively [54,55]. In *D. melanogaster* models, disrupting the function of mitochondrial complex I through genetic manipulation (for example, using mutation models of the *PINK1* or *parkin* genes) can simulate the pathological features of PD. *D. melanogaster* exhibits phenotypes such as dopaminergic neuron-specific loss, motor dysfunction, and mitochondrial morphological fragmentation, directly demonstrating that the failure of the mitochondrial quality control system leads to selective neuronal fragility [56]. However, overexpression of *PINK1* offsets the neurotoxicity of mHtt in HD, reduces the formation of mitochondrial aggregates, partially restores mitochondria, and ensures the integrity of neurons and the survival of adult *D. melanogaster* [57]. In *C. elegans*, partial functional loss of the complex I subunit NDUF-7 causes dopaminergic neurodegeneration in a dopamine neuron subtype-specific manner, and mitochondria-targeted antioxidants can effectively salvage this neurodegeneration [58]. Knockdown of the oxidative stress-responsive serine-rich protein 1 (*OSER1*) regulated by FOXO can lead to mitochondrial fragmentation, mitochondrial gene transcription alterations, reduced ATP, and shortened lifespan in *C. elegans*, *D. melanogaster*, and *B. mori* [59]. Knockout of mitochondrial transcription factor A (*BmTfam*) in *B. mori* can reduce the amount of mtDNA and induce growth retardation in the larval stage. Moreover, the expression of BmTFAM can rescue the reduction in mtDNA copy number and the increase in mtDNA nucleotides in human HeLa cells caused by *Tfam* knockdown. Therefore, BmTFAM can compensate for the function of human TFAM in HeLa cells, indicating that the mitochondrial function of TFAM is highly conserved between *B. mori* and humans [60]. ATAD3A is a mitochondrial membrane protein. In the *B. mori* model, knockout of *BmATAD3A* hinders mitochondrial activity and ATPase synthesis, inhibits the mitochondrial oxidative phosphorylation pathway, and thus significantly affects mitochondrial metabolism, body weight, and survival rate [61]. Knockout of the mitochondrial phosphatase *PTPMT1* gene can lead to embryonic death, developmental arrest and third-age death in *B. mori*. Therefore, *PTPMT1* is crucial for early development [62].

### 2.3. Axonal Transport Defects and Loss of Synaptic Function 

In the pathological cascade reaction of neurodegenerative diseases, axonal transport defects and the resulting loss of synaptic function are generally regarded as the core links driving the early symptoms of the disease and the disintegration of neural networks. Neurons are highly polarized cells, and their functional integrity is extremely dependent on the efficient operation of axons as a “lifeline”. Axonal bidirectional transport mediated by motor proteins (such as kinesin and cytoplasmic dynein) is responsible for transporting cargo synthesized by the cell body—including mitochondria, signaling molecules, and synaptic vesicles—to the distal presynaptic terminal, and for transmitting retrograde signals, such as neurotrophic factors, back to the nucleus. When this sophisticated system malfunctions due to genetic mutations, aggregation of toxic proteins, or energy crises, synapses are the first to be damaged. Insufficient mitochondrial supply can lead to local synaptic energy depletion and calcium homeostasis imbalance, while the interruption of protein and lipid supply necessary for the release and recycling of neurotransmitters will directly weaken the transmission efficiency and plasticity of signals. This long-term “nutrient deprivation” eventually leads to the atrophy of synaptic structures, functional degeneration, and even complete loss. It is worth noting that this process often occurs before the death of the neuronal cell body, explaining why many neurodegenerative diseases present with significant cognitive or motor dysfunction at an early stage. Numerous studies on model organisms have jointly confirmed that axonal transport is the lifeline for maintaining synaptic health. The collapse of this system is a key event that bridges the past and the future in the process of neurodegeneration, providing a crucial perspective for understanding disease mechanisms and developing early intervention strategies.

In the *D. melanogaster* model, overexpression of human Tau protein mimics the pathology of AD. Overly phosphorylated Tau forms neuronal fibrillary tangles, leading to instability in the microtubule structure and directly hindering the forward movement of transport motors, causing “traffic jams” for goods such as mitochondria in axons. This “imbalance” begins with a cascade of events at the molecular level. Under physiological conditions, Tau protein binds to tubulin through its repetitive domain to stabilize microtubules. However, site-specific hyperphosphorylation (such as Ser262 and Thr231) greatly reduces the affinity of Tau for microtubules, causing it to detach from the microtubule surface and making the microtubules lose their stabilizing factors and tend to depolymerize [63]. The detached phosphorylated Tau can occupy the binding sites on the surface of microtubules and directly interfere with the advancement of motor proteins [64]. It is worth noting that the presence of a large amount of phosphorylated Tau on microtubules can significantly alter the local transport kinetics of motor proteins [65]. Ultimately, the stagnation of organelles such as mitochondria triggers a local energy crisis, and energy exhaustion can further inhibit phosphatase activity and intensify Tau phosphorylation, creating a vicious cycle [66]. Therefore, the “traffic jam” is the result of the combined effect of chemical modification instability, physical space obstruction, and energy metabolism disorder.

*D. melanogaster* overexpressing Tau exhibits progressive impairments in learning behavior and motor coordination [67]. Furthermore, alterations in the expression of members of the *D. melanogaster* β-amyloid precursor protein (APP) gene family can also lead to axonal transport defects and affect synaptic plasticity [68]. In *C. elegans*, when mutations occur in the kinesin UNC-116, axons degenerate and the bidirectional transport of the dense core vesicles is greatly impaired, resulting in severe motor defects in *C. elegans*. This is because axonal transport is interrupted, resulting in the inability of presynaptic vesicles and proteins to aggregate and be maintained as usual, and the failure of synaptic signal transmission, thereby making the body of *C. elegans* stiff and difficult to move [69,70]. Kinesin-3 UNC-104 also has a similar function [71]. At present, there is no direct evidence indicating a connection between axonal transport defects, synaptic dysfunction, and neurodegenerative performance in *B. mori*. However, in studies using its complex olfactory neural circuits, neural dysfunction can lead to olfactory dysfunction [72], and the establishment of synaptic specificity can affect the olfactory local interneurons [73], so it is speculated that there is a connection between synaptic function and neural function. This implies that *B. mori* has considerable research advantages and discovery space in this aspect.

In addition to protein homeostasis imbalance, mitochondrial dysfunction, and axonal transport defects, invertebrate models can also be employed to explore other evolutionarily conserved pathogenic mechanisms. Neuroinflammation can be modeled in *D. melanogaster* through the Toll/NF-κB pathway to mimic glial-mediated innate immune responses, and studies have shown that this pathway regulates lifespan and healthspan in neuroendocrine cells and neuroblasts [74]. RNA metabolism and stress granules can be tracked in *C. elegans* and *D. melanogaster* through conserved RNA-binding protein networks, with ribonucleoprotein granules playing central roles in mRNA degradation, localization, and translational regulation [75]. Nucleocytoplasmic transport defects have been successfully recapitulated in *D. melanogaster* models of C9orf72, where expression of GGGGCC repeat expansions leads to nucleoporin degradation and impaired nucleocytoplasmic transport [76]. Lysosomal dysfunction and ER stress can be rapidly dissected through genetic screening: VAP protein deficiency in *D. melanogaster Vap33* mutants results in lysosomal acidification defects and impaired autophagic degradation [77], while *RNF-5* inactivation in *C. elegans* promotes *IRE-1*-independent ER stress resistance through enhanced autophagy [78]. Phase separation has been preliminarily shown to regulate protein condensation in *D. melanogaster* models, where cadmium-induced neurodegeneration is associated with impaired RNP formation and liquid–liquid phase separation [79]. Synaptic homeostasis serves as a sensitive readout of early neuronal dysfunction in *C. elegans*, and the Turing mechanism can stably control synaptic density during *C. elegans* growth [80]. The high conservation of these mechanisms in invertebrates further underscores the broad applicability and complementary value of the three models for multi-dimensional mechanistic dissection of neurodegenerative diseases.

## 3. Case Studies: Interpreting the Stories of Invertebrates in Various Neurodegenerative Diseases (AD, PD, and HD)

### 3.1. AD

AD is neuropathologically characterized by the abnormal deposition of Aβ in the brain, forming senile plaques, and the hyperphosphorylation of Tau protein, forming neurofibrillary tangles. The pathogenesis is complex; the prevailing view is that an imbalance between Aβ production and clearance serves as the initiating trigger, which in turn sets off a cascade of downstream events, ultimately leading to progressive cognitive decline. Research on AD in *C. elegans* began with the discovery of a homologous gene. In 1995, Levitan and Greenwald found that the *sel-12* gene in *C. elegans* was highly similar in sequence to *S182* (i.e., the presenilin gene *PSEN1*), and this gene was involved in *lin-12*-mediated signaling [81]. Knockout of the *PSEN1* gene in mice led to severe developmental defects, including impaired Notch signaling and embryonic lethality, which phenocopied the double mutant of *sel-12* and *hop-1* observed in *C. elegans*, further demonstrating functional conservation [82]. Furthermore, common marmoset models carrying *PSEN1* mutations generated by gene-editing technologies exhibited some AD-related biochemical changes [83]. Because *C. elegans* does not endogenously express Aβ, Link transferred the human β-amyloid peptide into its muscle cells for expression. These transgenic animals exhibited obvious Aβ aggregation and toxic phenotypes, acting as an early classic model. Although this model expresses Aβ in non-neuronal tissues, it demonstrated that *C. elegans* can reproduce the core pathological features of AD: protein aggregation and related toxicity [84]. Then, Fire et al. discovered that injecting double-stranded RNA (dsRNA) into *C. elegans* could produce a much stronger gene-silencing effect than single-stranded RNA (whether the sense strand or the antisense strand). This phenomenon is called RNA interference (RNAi) [27]. This landmark discovery laid the theoretical foundation for a powerful gene function research tool for more than two decades thereafter, enabling researchers to precisely manipulate the expression of specific genes and thereby explore the molecular mechanism of AD in a systematic way [27].

Since the beginning of the 21st century, researchers have constructed multiple *C. elegans* AD models to study the molecular mechanism of AD pathogenesis. These include models for studying the toxic mechanism of Aβ, the toxic function and mechanism of Tau protein, the function of APP, and the function of progerin, etc. Link published a review systematically summarizing the construction logic of *C. elegans* and *D. melanogaster* AD models and their revelation of the role of Aβ and Tau in the etiology of AD [85]. Later, Mhatre et al. also published a review of the same name in which the model construction and related studies of AD in *C. elegans* and *D. melanogaster* were further summarized [28]. The perspectives of these two articles are quite similar. Link’s article is pioneering, while the other article summarizes the research of the subsequent nearly ten years and provides a more comprehensive summary of transgenic AD models in invertebrates. Alvarez et al. also specifically reviewed the modeling of AD in *C. elegans* [86]. Compared with the previous two reviews, this review focused more on *C. elegans*, with a more concentrated target. These models have expanded the etiology of AD from merely “protein aggregation” to multi-dimensional cytopathological processes involving protein homeostasis and autophagy mechanisms, mitochondrial dysfunction, and endoplasmic reticulum stress.

Furthermore, a large number of traditional Chinese medicine extracts and natural products have been tested in the *C. elegans* system in order to search for new drugs and therapies for AD. For instance, Hernández-Ayala et al. found that quinoline derivatives exhibit diverse biological activities and can serve as multifunctional antioxidants against AD [87]; Wang et al. reported that *Morus nigra* fruit extract reduces Aβ oligomer deposition and ROS generation in *C. elegans* and activates the insulin DAF-16 signaling pathway to alleviate AD-like symptoms, exhibiting potential anti-AD effects [88]. There are numerous studies of this type, and no systematic conclusion has yet been reached. However, while identifying potential intervention targets, the general understanding of AD pathogenesis has been further deepened: (1) Toxicity stems from protein aggregation, but aggregation is not the entirety of pathology. (2) The integration of cellular stress responses is a key node in pathological progression. (3) Aging is the most significant risk factor for neurodegenerative diseases. *C. elegans* has become an important model for studying the molecular mechanism of AD and seeking new treatments.

*D. melanogaster* can be introduced into research on AD due to its natural advantage that nearly 70% of human disease-related genes have clear homologs in this species [89]. Using the GAL4/UAS expression system, any vertebrate gene can be expressed in specific tissues and cells of *D. melanogaster* [90]. As early as 1989, Rosen et al. indicated the existence of a gene encoding a human β-amyloid precursor protein (APP) in *D. melanogaster*, laying the foundation for future transgenic models [91]. Then, Kim et al. proposed that the lack of the *APPL* gene, the *D. melanogaster* homolog of human APP, leads to behavioral deficits, suggesting its role in brain function [92]. This study also suggested a possible direction for using this species to study AD [92], and wild-type and mutant human *APP* were expressed in tissue culture cells and transgenic species. Fossgreen et al. demonstrated the presence of γ-secretase activity in *D. melanogaster* and showed that full-length expression of *APP* causes the “bubble wing” phenotype [93].

There are two main types of AD models in the species. The first is based on Aβ toxicity, as represented by the following examples. In 2004, Iijima et al. reported that the expression of human Aβ42 in the *D. melanogaster* brain would lead to age-dependent learning deficits, amyloidosis, and neurodegeneration [94]. However, expression of Aβ40 only caused learning deficits without other obvious pathological changes [94]. In the same year, Finelli et al. established an independently validated Aβ42 toxicity model in *D. melanogaster* and found that *neprilysin* gene mutations could regulate the toxicity phenotype by altering the level of Aβ42 peptide [95]. These two representative works demonstrate that the species can reproduce the core pathology of Aβ-induced neurodegeneration. The second main model of AD is based on Tau protein. According to Wittmann et al., *D. melanogaster* individuals expressing wild-type and mutant human Tau proteins exhibited age-dependent neurodegeneration, motor deficits, and shortened lifespan but did not show typical neurofibrillary tangles, suggesting that Tau toxicity can occur independent of tangle formation [96].

Later, *D. melanogaster* models were systematically used to analyze the toxic mechanisms of Aβ and Tau, with a focus on the following aspects. The first is mitochondrial dysfunction. Varte et al.’s review systematically summarized the contributions of *D. melanogaster* to the study of mitochondrial dysfunction at multiple levels, ranging from oxidative stress and calcium homeostasis dysregulation to mitochondrial autophagy and mitochondrial fusion/division [97]. The second is neuroimmune interaction and the gut–brain axis. Arora et al. found that excessive activation of the NF-κB pathway mediated by *Pirk* can induce age-dependent neurophenotypes, and gut-specific knockdown of *Pirk* leads to the premature appearance of neurophenotypes, suggesting that intestinal immunity plays an important role in the early stage of neurodegeneration—the brain and gut ecosystems are associated with neurodegeneration [98]. The bidirectional relationship between Tau protein aggregation and gut microbiota dysbiosis in *D. melanogaster* was also explored by Alves et al. [99]. Parvez et al. further summarized the unique advantages of *D. melanogaster* in the analysis of the brain–peripheral immune axis [100]. The third is testing the effectiveness of potential therapeutic drugs and dietary plans. Bhatnagar et al. found that the Tip60 HAT activator can prevent neuronal defects in the *D. melanogaster* model of AD, reduce lethality, and significantly restore the expression of suppressed neuroplasticity genes in the brain [101]. According to Tan et al., the disruption of the gut microbiota can also affect the pathogenesis of AD. In the *D. melanogaster* model, *Lactobacillus plantarum* DR7 can ameliorate the effects of AD [102]. In addition, Leventhal et al. integrated data from proteomics, metabolomics, genomics, and lipidomics and discovered how HNRNPA2B1 and MEPCE enhance the toxicity of Tau protein, and the regulatory factors CSNK2A1 and NOTCH1 related to DNA damage repair in AD-associated neurons were screened out [103]. This type of work pushed the *D. melanogaster* model from the previous “targeted validation” to “systematic discovery”.

**Figure 2:**
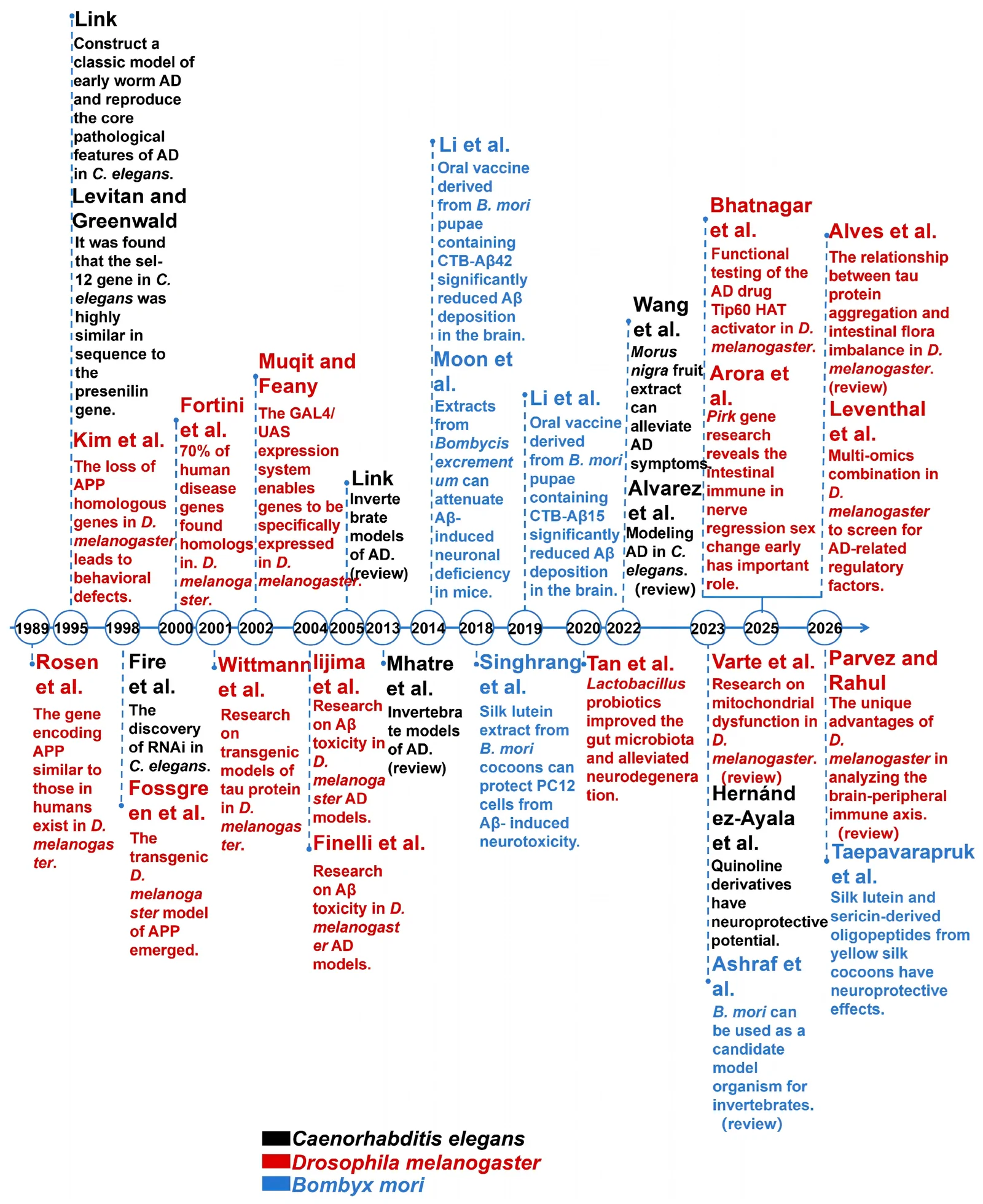
A brief introduction to research progress on AD in three invertebrates [7,27,28,81,84,85,86,87,88,89,90,91,92,93,94,95,96,97,98,99,100,101,102,103,104,105,106,107,108].

In 2014, Minho et al. found that extracts from *Bombycis excrementum* could significantly improve Aβ oligomer-induced memory impairment, neuronal loss, microglial proliferation, and astrocyte proliferation in mice [104]. Afterwards, the Singhrang team found that silk lutein extract from *B. mori* cocoons could be activated by reducing ROS production and inhibiting the mitochondrial apoptotic pathway and MAPK pathway and could protect PC12 cells from Aβ-induced neurotoxicity [105]. Coincidentally, Taepavarapruk et al. discovered that silk lutein and sericin-derived oligopeptides from yellow silk cocoons also have significant and overlapping neuroprotective effects in rodent models of Aβ-induced and age-related cognitive decline [106]. These studies provide empirical evidence for the potential application of traditional medicine in the prevention and treatment of AD. In addition, Li’s team has demonstrated that oral vaccines derived from *B. mori* pupae containing cholera toxin B subunit (CTB)-Aβ42 and CTB-Aβ15 significantly reduce Aβ deposition in the brain and improve cognitive function in transgenic AD mouse models, suggesting that *B. mori*-based vaccines hold promise for clinical application in the prevention of AD [107,108]. The role of *B. mori* in AD research is also undergoing a transformation from “drug resources” to “disease models”. *B. mori* was considered an emerging invertebrate model organism that could be used for human health and disease research [7]. A brief introduction to the research history of AD in three invertebrates is shown in Figure 2.

The three invertebrate models exhibit complementary strengths in AD research. Owing to its genetic tractability and the RNAi platform, *C. elegans* enables rapid gene-to-phenotype validation, making it a preferred system for conserved pathway identification and high-throughput target screening. *D. melanogaster*, with its GAL4/UAS system for spatiotemporal gene control and its rich behavioral repertoire, extends AD modeling to the system levels, providing unique opportunities to dissect neuroimmune interactions and the gut–brain axis. *B. mori* offers a distinct translational dimension: its extracts, sericin-derived peptides, and oral vaccines have shown neuroprotective effects, and it is increasingly recognized as an alternative invertebrate model. However, mechanistic understanding of the *B. mori* model remains less developed than that of the other two organisms, and the molecular relevance of its protein aggregation phenotypes to AD pathology requires further validation.

### 3.2. PD

PD is neuropathologically characterized by the progressive loss of dopaminergic neurons and the abnormal aggregation of α-synuclein in the remaining neurons. Its pathogenesis involves a complex interplay of multiple factors, including α-synuclein aggregation and toxicity, mitochondrial dysfunction, and impaired protein clearance pathways. In 2003, wild-type and A53T mutant human α-synuclein were introduced into *C. elegans* through genetic modification by Lakso et al. [109]. The results showed that human α-synuclein caused neuronal and behavioral abnormalities in *C. elegans* dependent on expression in specific neuron subtypes [109]. This work established a model of dopaminergic neuron degeneration and behavioral defects induced by α-synuclein in *C. elegans*, laying a solid foundation for the subsequent in-depth analysis of the pathological mechanism of PD. With the continuous discovery of PD pathogenic genes, the *C. elegans* PD model entered a systematic development stage. Cooper et al. and Chakraborty et al. found that the deletion of *pink-1* and *pdr-1* genes can cause phenotypes such as abnormal mitochondrial morphology (increased fragmentation), decreased oxidative phosphorylation rate, and reduced ATP levels and exhibit dopaminergic neuron loss and dopamine-dependent behavioral deficits [110,111]. The PD mechanisms related to oxidative stress were systematically reviewed by Sudipta et al., with particular emphasis on the evolutionary conservation of the Nrf2 homolog and the roles of PD-associated proteins (Parkin, DJ-1, and PINK1) in neurodegeneration [112]. Naidoo et al. discovered that miR-71 (an microRNA known to affect stress resistance and prolong life) could protect dopaminergic neurons in the LRRK2-induced *C. elegans* model of PD by regulating *tir*-1/SARM-1 [113]. The significant role of miRNA in regulating the toxicity of LRRK2 protein was revealed [113]. During the same period, neurotoxin exposure models were also developing rapidly. 6-hydroxydopamine (6-OHDA), MPTP, paraquat, and rotenone have been confirmed by da Silva et al. to induce progressive loss of dopaminergic neurons and decrease the survival rate in this species [114]. This provides an important supplement to the research on environmental factors in the pathogenesis of PD. Similar to AD, *C. elegans* can also be used as a tool to screen for drugs that are beneficial to the symptoms or disease progression of PD. For example, Redl et al. found that spiraeoside and miquelianin from *Filipendula ulmaria* effectively reduced the fluorescence intensity of α-synuclein in the NL5901 *C. elegans* PD model [115]. They simultaneously enhanced heat stress tolerance and inhibited the expression of pro-inflammatory cytokines in human microglia, highlighting the potential of a combined therapeutic strategy that simultaneously targets protein homeostasis and neuroinflammation [115].

Pioneering work by Feany and Bender introduced *D. melanogaster* as a model for PD by expressing A30P and A53T mutant human α-synuclein in the nervous system [116]. This study showed that transgenic *D. melanogaster* recapitulated key features of human PD, including adult-onset dopaminergic neuron loss, α-synuclein aggregation, and age-dependent motor deficits. Subsequent studies have indicated that the A53T mutant is generally associated with more severe pathology than A30P. This model has since served as a valuable platform for studying PD pathogenesis and therapeutic screening [116]. Similar to the *C. elegans* PD model, the successive discovery of PD pathogenic genes also brought the *D. melanogaster* PD model into a new stage of development, which includes multiple pathogenic genes such as *PINK1*, *DJ-1*, and *LRRK2*. For instance, researchers constructed multiple models of *PARKIN* and *PINK1* gene knockout or knockdown in this species. These models exhibited astonishing phenotypic consistency: severe mitochondrial morphological abnormalities, flight muscle degeneration, male infertility, dopaminergic neuron deficiency, and motor dysfunction. The more crucial finding was that age-dependent mitophagy exists in this species [117,118,119]. This discovery established mitochondrial dysfunction as the central link in the pathology of PD and pointed out the direction for subsequent mammalian research. *PARKIN* knockout mice were resistant to weight gain under a high-fat diet but exhibited glucose intolerance and insulin resistance. In addition, *PARKIN* deficiency was associated with the development of various cancers according to Paul et al. [120]. At the clinical level, Puschmann et al. found that *PARKIN* and *PINK1* were well-established causative genes for early-onset PD [121]. Notably, the dependency on the *PINK1*/*Parkin* pathway varied significantly across species. A particularly critical finding by Chen et al. was that *PINK1* was selectively expressed in the primate brain, but not in rodent or porcine brains, suggesting that *PINK1* expression might be primate-specific [122]. Similarly, the systematic development of neurotoxin-induced models advanced in parallel. Rotenone, paraquat, and 6-OHDA exposure could induce dopaminergic neuronal degeneration, dyskinesia, and α-synuclein aggregation in *D. melanogaster* [123,124], providing important evidence for studying the role of environmental factors in sporadic PD. The unique advantages of *D. melanogaster* also make it an ideal tool for studying neuroimmune interactions and the gut–brain axis. Researchers are using this model to explore complex and conserved molecular mechanisms and screen drugs effective for PD [100,123,125,126,127]. Moreover, the focus of *D. melanogaster* PD research has gradually expanded from the simple death of dopaminergic neurons to aspects such as the role of glial cells and the terminal manifestations of intercellular interaction networks. For instance, Pak et al. discovered that specifically reducing the phosphatidylserine synthase gene *Pss* in cortical glial cells could lead to dopaminergic cell death [128]. Meimoun et al. used the species to simulate *PLA2G6* (PARK14)-related neurodegenerative changes [129]. The results showed that although *PLA2G6* mutations clinically present as early-onset Parkinson’s syndrome, the *D. melanogaster* model indicated that specific knockdown of *iPLA2-VIA* in GABAergic neurons was sufficient to cause severe motor deficits and shortened lifespan, while knockdown only produced a weak effect in dopaminergic neurons [129].

Tabunoki et al. systematically discussed the possibility of using *B. mori* as a human disease model. Moreover, the study found that a spontaneous mutant strain of *B. mori* had a significant molecular network overlap in the gene expression profile with that of PD patients [130]. This provides a genetic basis for the study of PD using endogenous mutations in *B. mori*. Afterwards, a landmark study was published. Song et al. treated the species with the neurotoxin MPTP and successfully established a PD model [131]. They conducted a systematic assessment from multiple dimensions, including growth conditions, behavior, physiological and biochemical aspects, and gene regulatory pathways. This model reproduced the core pathological features of human PD. Moreover, the clinical PD treatment drugs levodopa and carbidopa had significant therapeutic effects on the *B. mori* model, verifying the clinical relevance of the model. On this basis, the screening team also found that *Lycium barbarum* polysaccharide (LBP) could not only improve various pathological features of PD but also have outstanding effects on oxidative damage repair [131]. This work runs through the entire chain of “construction of animal models of human diseases–treatment validation–screening of new drugs”. Furthermore, Zhu et al. first established a *B. mori* model of PD via chronic 6-OHDA feeding. The model displayed PD-like phenotypes, including motor dysfunction, dopaminergic neuron loss, and decreased dopamine levels. Proteomic and metabolomic analyses further revealed conserved pathogenic pathways, supporting *B. mori* as a feasible and valuable model for PD mechanism studies [132]. In 2024, Zhang et al. published another significant study [133]. After treating *B. mori* ovarian BmN cells with rotenone and conducting transcriptome sequencing, they found that rotenone led to a significant reduction in mitochondrial membrane potential, thereby inducing mitochondrial autophagy. The PINK1/Parkin pathway played a key role in this process. Further research exposed *B. mori* to 50 μg/mL rotenone and observed unique motor dysfunction [133]. This provided a new basis for investigating the environmental etiology of sporadic PD. In addition, a key uric acid synthesis pathway was identified in a unique mutant *B. mori* model of PD. By analyzing the op mutant, Tabunoki et al. found that the expression of genes involved in the DJ-1-related uric acid synthesis pathway was significantly downregulated, along with a marked reduction in brain tyrosine hydroxylase mRNA levels and increased sensitivity to oxidative stress [134]. This supported the use of *B. mori* as an effective animal model for studying the molecular mechanisms of PD.

In PD research, *C. elegans* has enabled critical discoveries ranging from α-synuclein toxicity to the PINK1/Parkin mitochondrial pathway, and its genetic tractability makes it particularly suitable for target identification; however, its simple neural circuitry cannot recapitulate the complex dopaminergic regulatory network. *D. melanogaster* has advanced PD research to the systems level, uncovering new roles for glial cells and GABAergic neurons in PD pathology and establishing mitochondrial quality control as a central pathogenic mechanism. Nevertheless, differences remain between *D. melanogaster* and humans in terms of dopaminergic system architecture and metabolic regulation. *B. mori* bridges the translational pipeline from model construction and therapeutic validation to novel drug screening through toxin-induced models (MPTP, 6-OHDA, and rotenone) combined with clinical drug validation and shows particular value in studying environmental etiology and metabolism. However, its mechanistic depth currently lags behind that of *C. elegans* and *D. melanogaster*.

### 3.3. HD

HD is caused by CAG repeat expansion in the *HTT* gene, leading to mutant HTT aggregation, which triggers mitochondrial dysfunction, transcriptional dysregulation, and impaired autophagy, culminating in selective neurodegeneration and progressive motor, cognitive, and psychiatric symptoms. One of the earliest HD models of *C. elegans* was the Htn-Q150 model, which was established by Faber et al. in 1999 and expresses human huntingtin fragments containing 150 polyglutamines in ASH sensory neurons [135]. This model could lead to the formation of HTT aggregates and neurodegeneration [135]. Later, according to Parker et al., the results of the specific expression of the *htt* gene in mechanosensory neurons showed that compared with the expression of short normal (19 Glns) repeat sequences, the expression of 57 amino acids (Htt57) of HTT proteins containing the long polyglutamine tail (88 and 128 Glns) led to the formation of aggregates in axons [136]. Moreover, this change was associated with axon abnormalities rather than cell death. The hint was synaptic connection loss rather than neurodegeneration [136]. In the body wall muscle cells, polyglutamine was repeatedly expressed in the form of a green fluorescent protein (GFP) fusion protein. The expansion of polyglutamine aggregates with 82 residues increased the brightness and quantity of fluorescent foci within myofibrils and affected the lifespan of *C. elegans* [137,138]. When 40 glutamine repeat sequence fragments (Q40) fused with yellow fluorescent protein (YFP) were expressed in the muscle of *C. elegans* strain AM141, this transgenic model exhibited fluorescent protein aggregates in the body wall muscles. Moreover, as *C. elegans* aged, it gradually became paralyzed and eventually died at approximately 14 days of age [138,139]. These studies indicated that longer polyQ repeats tend to cause more severe aggregation and toxicity, but polyQ of the same or similar lengths produced different pathological consequences in different neuronal types, suggesting that the cellular environment also played an important regulatory role in polyQ toxicity.

In 1998, Jackson et al. constructed HD models carrying CAG repeat sequences of different lengths—Htt-Q2, Htt-Q75, and Htt-Q120—in *D. melanogaster*, demonstrating that the length of CAG repeat sequences was positively correlated with the progressive degeneration of photoreceptor neurons [140]. Then, Steffan et al. reported another significant advancement—the Httex1pQ93 model, jointly expressing 93 CAG repeat sequences and huntingtin exon 1 protein (Httex1p) [141]. The result led to the degeneration and shortened lifespan of photoreceptor neurons [141]. In the 21st century, research on the *D. melanogaster* HD model has continuously improved. The Htt-Q128 model (containing 128 repeated polyQ fragments) was constructed by Lee et al. and found to cause ocular roughness, degeneration of photoreceptor neurons, impaired motor function, and shortened lifespan, providing a new tool for behavioral assessment [142], while the full-length HD model Htt128QFL (containing 128 CAG repeat sequences) was constructed to express the full-length HTT rather than just the N-terminal fragment. Romero et al. reported that this model was more toxic and led to a significant shortening of the *D. melanogaster* lifespan, being closer to the pathological features of human HD [143]. Schulte et al. created an Htt-RFP model carrying a monomeric red fluorescent protein (mRFP) tag, which contains 138 polyQs and caspase-6 cleavage fragments [144]. In vivo imaging tracking of the distribution and aggregation dynamics of HTT proteins was achieved. This tool was of great value for understanding the formation kinetics of aggregates and screening compounds that inhibit aggregation [144]. Furthermore, Krench and Littleton found that the aggregates formed by pathogenic HTT could rapidly form within a few hours, and these aggregates physically blocked the transport of multiple organelles along the axon [145]. A key mechanism of neurodegeneration in HD was revealed—axonal transport disorder [145]. Meanwhile, using *D. melanogaster* as a drug screening platform, multiple compounds displayed verifiable neuroprotective activity. According to Chongtham et al., curcumin significantly improved the disease symptoms of HD by inhibiting cell death and could be the key to suppressing the progression of HD with minimal side effects [146]. Teseo et al. reported that extracts of *G. sinense* and *P. notoginseng* exhibited neuroprotective effects in the *D. melanogaster* Htt-Q128 model and prolonged the lifespan of senescent individuals [147]. In the Htt128QFL model, Bolshakova et al. found that fullerenols could exert antioxidant effects and improve neurodegenerative processes [148], while Cristo et al. found that meldonium could improve mitochondrial dysfunction [149].

To date, a direct *B. mori* model of HD recapitulating neural pathology has not yet been established. The existing evidence is limited to indirect similarities between mechanisms in the domains of oxidative stress and autophagy dysfunction [52,59]. These conserved pathways may provide a possible perspective for future research on HD in *B. mori*.

In HD research, a “dose–effect” model has been established in *C. elegans* in which polyQ length is positively correlated with toxicity, revealing the modulatory role of the cellular environment on toxicity, but it could not recapitulate complex motor and cognitive deficits. *D. melanogaster*, through iterative modeling from fragment to full-length constructs (Htt-Q120 → Httex1pQ93 → Htt128QFL), has enabled the modeling of pathology, behavior, and multi-dimensional drug responses, particularly advancing our understanding of aggregation dynamics through studies of axonal transport blockage and in vivo imaging, although its simulation of fully explicit disease features remains limited. So far, no direct *B. mori* HD model exists; evidence is limited to parallels between oxidative stress and autophagy. Systematic pathogenic gene manipulation and quantitative phenotyping in this model are not yet mature. While *C. elegans* and *D. melanogaster* have established a strong consensus in the field, research in *B. mori* is still in its early stages and awaits more direct genetic evidence.

Furthermore, we have summarized the recent advances since 2020 in neurodegenerative disease research across these three species (Supplementary Table S1; Refs. [150,151,152,153,154,155,156,157,158,159,160,161,162,163,164,165,166,167,168,169,170,171,172,173,174,175,176,177,178,179,180,181]).

In summary, the three models each possess distinct advantages, collectively delineating a complementary pathway from mechanistic discovery to preclinical validation. However, none of them can faithfully recapitulate the age-dependent slow progression or the complex cerebrovascular microenvironment of human neurodegenerative diseases. Therefore, a tiered integration strategy—employing *C. elegans* and *D. melanogaster* for initial screening, *B. mori* for translational validation, and mammalian systems for confirmation—may be adopted to fully leverage the strengths of each model and facilitate the effective translation from mechanistic insights to clinical intervention.

## 4. Discussion and Prospects: Potential Implications of Existing Research for the Prevention and Treatment of Future Neurodegenerative Diseases

### 4.1. Implications for Future Prevention and Treatment in Terms of the Material Basis

Thus far, no long-term effective treatment for neurodegenerative diseases has been explored. Therefore, the development of new drugs or new treatment methods is very necessary. However, the research results show that different pathogenic genes, proteins and pathological pathways are involved in different neurodegenerative diseases. For instance, the study of *C. elegans* SKN-1/Nrf2 revealed that this transcription factor can protect against oxidative stress through different mechanisms [182]; research on the PINK1/parkin pathway in *D. melanogaster* indicated that mitochondrial dysfunction was an important factor in the degeneration of dopaminergic neurons [118]. Therefore, in drug screening and development for neurodegenerative diseases, it is crucial to understand the interactions among the pathways related to the same disease. Only by knowing how they interact can the corresponding targets be identified. Based on the research on *C. elegans*, *D. melanogaster*, and *B. mori* in AD, PD, and HD, the next generation of treatment strategies can shift from “clearing single proteins” to “repairing the system functions of the material basis”—maintaining lysosomal acidification, activating mitochondrial function, and restoring lipid transport between organelles.

### 4.2. Implications of Existing Treatment Strategies for Future Prevention and Treatment

The prevention and treatment of neurodegenerative diseases are major medical challenges because their pathogenesis is extremely complex. Current treatments can only provide limited relief and often bring adverse side effects. Therefore, we need to explore more effective and safer methods and drugs. Take AD as an example.

Natural plant products can be used in the prevention and treatment of AD. In the AD *D. melanogaster* model, although *Gardenia jasminoides* extract could effectively improve memory impairment and downregulate brain inflammatory responses, it failed to significantly extend lifespan or reduce Aβ deposition [183]. In contrast, the water extract of *Convolvulus pluricaulis* could not only significantly save the premature death caused by Tau protein and prolong life but also reduce the level of Tau protein and alleviated oxidative stress, which manifested as a decrease in ROS and lipid peroxidation (LPO) content and a reduction in Tau protein oxidative damage [184]. Luteolin also has antioxidant and anti-aggregation activities. While reducing LPO, it enhances SOD activity and inhibits the formation of Aβ plaques [185]. Quercetin can reduce Aβ toxicity in *D. melanogaster* with AD by regulating the expression of cyclin B [186]. The leaves of *Solanum macrocarpon* and *Solanum nigrum* also improved motor performance, memory index, and ROS levels in *D. melanogaster* with AD through neural enzyme modulatory and antioxidant mechanisms [187]. It is worth noting that curcumin promotes amyloid fibril conversion by reducing Aβ pre-fibrillar/oligomeric species and downregulating acetylcholinesterase *(AChE*) expression, thereby exerting neuroprotective effects [188]. The aqueous leaf extract of *Phyllantus amarus* plays a role in the treatment of AD by improving the survival rate and climbing activity of *D. melanogaster* with AD through anticholinesterase and antioxidant potential [189].

Drugs that act on other diseases can also impact the prevention and treatment of neurodegenerative diseases. Drugs that regulate the intestinal flora play a significant role in regulating the progression of AD [190,191], and the use of anti-aging drugs can delay the progression of AD [192].

Monotherapy for Aβ and tau has limitations. People are also turning their attention to multi-target methods, which are regarded as very attractive strategies for the design and discovery of AD drugs. There are methods involving phytochemicals, as well as methods involving heterocyclic compounds [87,193]. In particular, the integration of artificial intelligence will also significantly transform the existing therapeutic fields, accelerating target prediction and optimizing treatment plans, etc.

In the future, nanotechnology will also play an important role in the prevention and treatment of neurodegenerative diseases. Protein nanoparticle-induced osmotic pressure plays a key role in the pathogenesis of AD. Restoring intracellular osmotic pressure can serve as a potential target for developing new and effective therapeutic strategies for the disease [194]. Targeted oxidation can be achieved using multicomponent nanoscintillators. In the *C. elegans* AD model fed with nanoscintillators, the scintillators interact with Aβ in *C. elegans*, and then X-ray irradiation of the scintillators can reduce Aβ levels and extend life [195]. Chitosan nanoparticles loaded with tanshinone IIA (Tan IIA) may reduce paralysis and Aβ deposition in the *C. elegans* model of AD through the DF16/SOD-3 pathway and autophagy [196]. Fluorescence imaging technology is used to diagnose AD by detecting the accumulation of Aβ via carbon-dot–curcumin nanoparticle conjugation in *D. melanogaster* [197].

In the prevention and treatment of neurodegenerative diseases, dietary health and air quality cannot be ignored. Methylglyoxal (MG) is usually a precursor of advanced glycation end-products produced during cooking. Long-term dietary intake of MG promotes the accumulation of Aβ and disrupts autophagy in *C. elegans* to exacerbate the pathogenesis of AD [198]. Dietary intervention can improve cellular competition and motor ability in a model of AD in *D. melanogaster* [199]. Early and long-term exposure to di(2-ethylhexyl) phthalate (DEHP) increases Aβ toxicity in *C. elegans* AD models, and this toxicity is associated with the expression of the autophagy-related gene *bec-1* [200]. Silica nanoparticles, a wear component of tires, can cause a significant reduction in motor ability and neurodegeneration in models of *C. elegans* AD and PD. Aging and elevated temperature can exacerbate these neurodegenerative diseases [201].

When it comes to neurodegenerative diseases, the most urgent clinical revelation at present is that treatment needs to be moved forward to the pre-symptom stage, focusing on enhancing the “stress resilience” of neurons. This is precisely the greatest lesson that model organisms with an extremely short life cycle offer to humanity—to repair the quality control system of cells before degeneration becomes irreversible.

Despite the contributions of invertebrate models, several major questions remain—particularly regarding the translation of conserved mechanisms to human pathology and the selective vulnerability of specific neuronal populations. Emerging technologies offer solutions. CRISPR-based genome editing enables precise modeling of disease alleles, expanding genetic toolkits beyond classical mutants. Single-cell and spatial transcriptomics, combined with proteomics, can dissect cell-type-specific vulnerability and capture protein aggregation dynamics at the systems level. Meanwhile, AI-assisted drug discovery can integrate invertebrate high-throughput data with machine learning to accelerate compound screening. Critically, a future priority is the translational validation of invertebrate findings in mammalian systems—including humanized mice, iPSC-derived neurons, and organoids—to confirm therapeutic relevance. These approaches will transform invertebrate models from screening tools into integral components of a cross-species translational pipeline. A comparison of the three invertebrate models—including their limitations, suitable modeling processes, and processes that cannot be adequately modeled—is presented in Table 1.

## 5. Conclusions

In summary, *C. elegans*, *D. melanogaster*, and *B. mori* offer complementary advantages for studying conserved mechanisms of neurodegenerative diseases. The neural structure of *C. elegans* is relatively fixed, and its transparent body is convenient for imaging, making it suitable for analyzing the basic logic of sensory–motor conversion. *D. melanogaster* has a convenient genetic manipulation platform that can more precisely analyze complex physiological processes such as the brain–gut axis, making it suitable for studying the modular organization of behavioral regulation. The large ganglia of *B. mori* are relatively more distinct, providing unique anatomical advantages for subcellular morphological tracing. However, all three models have limitations in recapitulating slow progression and age-dependent pathology, cerebrovascular complexity, and higher-order cognitive dysfunction. Future research may adopt a tiered strategy—from invertebrate discovery to mammalian validation—supported by emerging technologies such as CRISPR screening, multi-omics, and AI-assisted drug discovery. The integrated use of these models will facilitate the translation of mechanistic insights into therapeutic strategies for neurodegenerative diseases.

## Figures and Tables

**Table 1:** A comparison of the characteristics of the three invertebrate models.

Invertebrate Model	*C. elegans*	*D. melanogaster*	*B. mori*
Neuron Count	302 neurons, 6393 chemical synapses, and 890 gap junctions	~2 × 10^5^; 3016 neurons and 548,000 synapses identified in the larval brain to date	Unclarified
Key Advantages	Genetic tractability, genome-wide RNAi screening, and optical transparency for in vivo imaging	GAL4/UAS system for spatiotemporal gene manipulation, rich behavioral paradigms	Large ganglia facilitating anatomical tracing, abundant biological materials, cost-effective and easy husbandry
Key Limitations	Limited behavioral repertoire, lack of myelin, lack of a cerebrovascular system	Dopaminergic system anatomy differs substantially from that of humans	Limited genetic tools, weak behavioral research foundation
Biological Processes Amenable to Study	Conserved signaling pathways, protein aggregation toxicity, development and aging, synaptic transmission	Neural circuits and behavior, neuroimmune interactions, brain–gut axis, learning and memory	Autophagy–lysosomal pathway associated with protein aggregation, screening for neuroprotective bioactive compounds
Processes That Cannot Be Adequately Modeled	Higher-order cognitive functions, adaptive immunity, systemic inflammation, blood–brain barrier permeability	Adaptive immunity, blood–brain barrier permeability, chronic disease progression	Higher-order cognitive functions, complex behavioral paradigms, adaptive immunity
Representative Research Tools	RNAi, CRISPR/Cas9, fluorescent labeling	GAL4/UAS system, RNAi, CRISPR/Cas9	Transgenic expression, RNAi, omics analysis
Research Cycle	2–3 weeks	~2 months	1–2 months

## Data Availability

The original contributions presented in this study are included in the article/Supplementary Materials. Further inquiries can be directed to the corresponding author(s).

## Supplementary Materials

The following supporting information can be downloaded at https://www.mdpi.com/article/10.3390/biology15161351/s1, Table S1: Significant Research Advances of Neurodegenerative Diseases in Three Species Since 2020.

## Abbreviations

*C. elegans*	*Caenorhabditis elegans*
*D. melanogaster*	*Drosophila melanogaster*
*B. mori*	*Bombyx mori*
AD	Alzheimer’s disease
PD	Parkinson’s disease
HD	Huntington’s Disease
UPS	ubiquitin–proteasome system
ALP	autophagy–lysosome pathway
UFP/MFP	unfolded or misfolded protein
Aβ	β-amyloid protein
HTT	huntingtin protein
Nd	naked pupa
ROS	reactive oxygen species
APP	β-amyloid precursor protein
6-OHDA	6-hydroxydopamine
LBP	*Lycium barbarum* polysaccharide
