# Supplementary material for: Revealing the Mechanisms of Alzheimer’s, Parkinson’s and Huntington’s Diseases Through Invertebrate Models
Source: Biology (Basel). 2026 Aug 10;15(16):1351. doi: 10.3390/biology15161351 (PMC13509541; doi:10.3390/biology15161351)
Supplement: Supplementary file 1 [file biology-15-01351-s001.zip › biology-4394200-supplementary.pdf]

Supplementary Table S1: Significant Research Advances in Neurodegenerative Diseases in Three Species Since 2020.

Disease type	Invertebrate model	Mode of action	Effect	References
AD	*C. elegans*	STUB1-VCP/p97 axis ubiquitinates and degrades full-length PINK1 to constrain its abundance.	Axis inactivation impairs mitophagy and memory, with its expression reduced in AD patient brains.	150
AD	*D. melanogaster*	USP11 deubiquitinates and stabilizes ITCH, and excess ITCH disrupts the Golgi and suppresses autolysosomal degradation.	The USP11-ITCH axis is a shared pathogenic pathway of AD and ALS, and targeting it alleviates neuronal proteotoxic injury.	151
AD	*D. melanogaster*	Secreted Aβ42 *Drosophila* model built to probe neurotoxicity via fluorescence, behavior and live imaging.	Secreted Aβ42 differentially damages neurons to trigger apoptosis/ferroptosis; ferroptosis inhibitors restore locomotor function.	152
AD	*C. elegans*	Spermine induces Tau/α-syn liquid condensates via electrostatic interactions for autophagic clearance.	Spermine blocks fibrillization and mitigates neurotoxicity in *C. elegans*.	153
AD	*C. elegans*	Hederagenin activates the PPARα/TFEB signaling axis to boost lysosomal autophagy flux.	Enhanced autophagy clears amyloid aggregates and relieves AD-related neuronal impairment.	154
AD	*B. mori*	Extracts from Penthorum chinense Pursh modulate iron metabolism and antioxidant pathways to suppress ferroptosis.	They alleviate neuronal damage and AD-related pathological phenotypes in cell and nematode models.	155

AD	*C. elegans*	Spautin-1 activates the PINK1-PRKN mitophagy pathway.	It eliminates damaged mitochondria and rescues learning deficits in AD models.	156
AD	*C. elegans*	Two evolutionarily conserved epigenetic factors remodel chromatin and disrupt metabolic and autophagy networks.	Their abnormal accumulation accelerates biomacromolecule damage, shortens organism healthspan and drives ageing progression.	157
AD	*C. elegans*	Ciliatoside A triggers PINK1/Parkin-dependent mitophagy to clear damaged mitochondria and simultaneously blocks the assembly and activation of the NLRP3 inflammasome signaling.	Dual regulation of mitophagy and NLRP3 inflammasome suppresses neuroinflammation, reduces neuronal loss and ameliorates Alzheimer's pathological phenotypes.	158
AD	*D. melanogaster*	Boost neuronal reticulophagy to clear accumulated toxic APP protein.	Reticulophagy activation alleviates aging defects and APP-related neurotoxicity.	159
AD	*D. melanogaster*	Novel cannabinol derivatives identified via fragment screening suppress oxytosis and ferroptosis.	These compounds exert neuroprotective effects for neurological disorder treatment.	160
AD	*D. melanogaster*	LAMP proteins bidirectionally regulate lysosomal pH with opposing functional effects.	Distinct LAMP variants exert contradictory impacts on lysosomal degradation homeostasis.	161
AD	*D. melanogaster*	APP participates in a conserved aging-related proteostasis regulatory network.	Maintaining APP function alleviates age-associated protein aggregation disorders.	162
AD	*D. melanogaster*	Pathogenic tau triggers TE-derived	Blocking TE dsRNA suppresses tau-mediated	163

		dsRNA accumulation to initiate neuroinflammation.	neurodegeneration.	
PD	*D. melanogaster*	Mitochondrial fumarate blocks Parkin-dependent mitophagy.	Accumulated fumarate aggravates mitochondrial damage and neurotoxicity.	164
PD	*C. elegans*	Nanoplastics disrupt mitochondrial function, disturb redox balance and promote toxic α-synuclein aggregation.	Nanoplastics aggravate motor defects and neuronal damage in *C. elegans* and human cell PD models.	165
PD	*D. melanogaster*	PINK1, Keap1 and Rtnl1 cooperate to initiate selective reticulophagy for ER turnover in developmental stages.	The triple regulatory axis sustains endoplasmic reticulum proteostasis and normal developmental progression.	166
PD	*D. melanogaster*	EndophilinA links neuronal calcium influx to synaptic autophagy; a Parkinson-related mutation disrupts this coupling.	Broken synaptic autophagy triggers synaptic dysfunction underlying Parkinson’s neurodegeneration.	167
PD	*D. melanogaster*	Loss-of-function SGIP1 mutation disrupts synaptic endocytosis–autophagy coupling and synaptic proteostasis.	It induces dopaminergic synaptic damage and recessive early-onset parkinsonism.	168
PD	*D. melanogaster*	Striatum-enriched CalDAG-GEFI directly binds the Roc-COR GTPase domain of LRRK2, catalyzes GDP-GTP nucleotide exchange, elevates the LRRK2 GTP-bound active	Excessive CalDAG-GEFI-mediated LRRK2 activation disrupts mitochondrial turnover, impairs synaptic autophagy, and drives dopaminergic neuron loss; inhibiting CalDAG-GEFI-LRRK2	169

		state, and boosts its downstream kinase and cytotoxic signaling pathways.	interaction alleviates LRRK2-linked Parkinson’s neurodegeneration in fly and mouse models.	
PD	*D. melanogaster*	Accumulated mitochondrial CISD1/Cisd inhibits PINK1/Parkin-dependent mitophagy.	Suppressing CISD1/Cisd genetically or pharmacologically restores mitophagy and alleviates neurodegeneration in PD models.	170
PD	*C. elegans*	Low-dose F-53B disrupts redox balance and promotes α-synuclein aggregation in *C. elegans*.	The pollutant accelerates aging and triggers Parkinson-like pathological and behavioral defects.	171
PD	*C. elegans*	Block ferroptosis signaling to counteract α-synuclein-triggered neuronal death.	Ferroptosis suppression alleviates synucleinopathy-associated neurodegeneration.	172
PD	*C. elegans*	Genetically modified *C. elegans* overexpress disease proteins to recapitulate neurodegenerative lesions.	This simple organism serves as a convenient tool to explore aging and neurodegeneration mechanisms.	173
PD	*C. elegans*	Engineer Hsp104 pore-loop residues to gain specific recognition and clearance of toxic α-synuclein.	Engineered variants eliminate α-syn aggregates without off-target cytotoxicity, alleviating Parkinsonian neurodegeneration.	174
PD	*C. elegans*	Gut microbiota secretes biofilm amyloid proteins that enter circulation and disturb neural proteostasis.	Microbial amyloids trigger systemic inflammation and promote progressive neurodegeneration.	175
HD	*C. elegans*	Mild inhibition of the V-ATPase subunit vha-6 triggers an	LySR elevates lysosomal proteolytic capacity, accelerates mutant HTT	176

		ELT-2-dependent lysosomal surveillance response (LySR) in *C. elegans*.	aggregate clearance in an HD worm model, and greatly prolongs organismal healthspan independent of the classic insulin longevity pathway.	
HD	*C. elegans*	Aging-induced USP4 stabilizes EPS8 to overactivate RAC-JNK/actin cascade and trigger pathological protein aggregation.	Blocking the conserved USP4-EPS8-RAC axis mitigates HD polyQ and ALS FUS/TDP-43 aggregation, restores neuronal function and prolongs lifespan in *C. elegans* and human cell models.	177
HD	*C. elegans*	Cold activates TRPA1-NHR-49-PA28 γ to enhance ubiquitin-independent proteasomal degradation.	This conserved pathway clears HD/ALS toxic protein aggregates and improves longevity across *C. elegans* and human cell models.	178
HD	*C. elegans*	Chloroplast SPP binds polyQ-HTT to block its aggregation independent of the proteasome/autophagy.	Plant SPP reduces toxic HTT aggregates and neurotoxicity in HD cell/worm models.	179
HD	*D. melanogaster*	Engineered hsPEX19-FV binds the mHTT N17 domain via a mutated α4 helix to block early aggregation, ATP-independent.	hsPEX19-FV relieves mHTT proteotoxicity in human cells, mouse striatal neurons and *D. melanogaster* HD models.	180
HD	*D. melanogaster*	Mutant HTT sequesters YY1 to boost PAPD5, degrade miR-7 and trigger TAB2-TAK1-JNK apoptosis.	The PAPD5 inhibitor BCH001 alleviates neuronal death across multiple HD models.	181
